# Cloning, Functional Characterization and Site-Directed Mutagenesis of 4-Coumarate: Coenzyme A Ligase (4CL) Involved in Coumarin Biosynthesis in *Peucedanum praeruptorum* Dunn

**DOI:** 10.3389/fpls.2017.00004

**Published:** 2017-01-17

**Authors:** Tingting Liu, Ruolan Yao, Yucheng Zhao, Sheng Xu, Chuanlong Huang, Jun Luo, Lingyi Kong

**Affiliations:** ^1^State Key Laboratory of Natural Medicines, Department of Natural Medicinal Chemistry, China Pharmaceutical UniversityNanjing, China; ^2^Institute of Botany, Jiangsu Province and Chinese Academy of SciencesNanjing, China

**Keywords:** 4-coumarate: CoA ligase, *Peucedanum praeruptorum*, biochemical characterization, biosynthesis mechanism, site-directed mutagenesis

## Abstract

Coumarins are the main bioactive compounds in *Peucedanum praeruptorum* Dunn, a common Chinese herbal medicine. Nevertheless, the genes involved in the biosynthesis of core structure of coumarin in *P. praeruptorum* have not been identified yet. 4-Coumarate: CoA ligase (4CL) catalyzes the formation of hydroxycinnamates CoA esters, and plays an essential role at the divergence point from general phenylpropanoid metabolism to major branch pathway of coumarin. Here, three novel putative 4CL genes (Pp4CL1, Pp4CL7, and Pp4CL10) were isolated from *P. praeruptorum*. Biochemical characterization of the recombinant proteins revealed that Pp4CL1 utilized *p*-coumaric and ferulic acids as its two main substrates for coumarin biosynthesis in *P. praeruptorum*. Furthermore, Pp4CL1 also exhibited activity toward caffeic, cinnamic, isoferulic, and *o*-coumaric acids and represented a bona fide 4CL. Pp4CL7 and Pp4CL10 had no catalytic activity toward hydroxycinnamic acid compounds. But they had close phylogenetic relationship to true 4CLs and were defined as 4CL-like genes. Among all putative 4CLs, Pp4CL1 was the most highly expressed gene in roots, and its expression level was significantly up-regulated in mature roots compared with seedlings. Subcellular localization studies showed that Pp4CL1 and Pp4CL10 proteins were localized in the cytosol. In addition, site-directed mutagenesis of Pp4CL1 demonstrated that amino acids of Tyr-239, Ala-243, Met-306, Ala-309, Gly-334, Lys-441, Gln-446, and Lys-526 were essential for substrate binding or catalytic activities. The characterization and site-directed mutagenesis studies of Pp4CL1 lays a solid foundation for elucidating the biosynthetic mechanisms of coumarins in *P. praeruptorum* and provides further insights in understanding the structure–function relationships of this important family of proteins.

## Introduction

Coumarins, which are widely distributed in plant families of Apiaceae, Fabaceae, Compositae, Rutaceae, Colanaceae, and Thymelaeaceae, have been reported to have various important therapeutic properties, such as anti-inflammatory (Witaicenis et al., 2013), antibacterial (Schinkovitz et al., 2003; Céspedes et al., 2006), anti-coagulant effects, anti-cancer (Wu et al., 2003) and anti-AIDS activities. The biosynthetic pathway of coumarin is a part of phenylpropanoid metabolism. In recent years, many enzymes involved in the biosynthesis of coumarins have been isolated, such as phenylalanine ammonia lyase (PAL), cinnamate 4-hydroxylase (C4H), 4CL, 4-coumaroyl CoA 2′-hydroxylase (C2′H) (Vialart et al., 2012), psoralen synthase, angelicin synthase (Larbat et al., 2007, 2009) and bergaptol *O*-methyltransferase (BMT) (Hehmann et al., 2004). L-phenylalanine is catalyzed by PAL to form *trans*-cinnamic acid, which is further converted to *p*-coumaric acid by C4H. *p*-Coumaric acid is then activated by a member of the 4CL family (4CL; EC 6.2.1.12) into *p*-coumaroyl CoA. CoA-ester intermediate is subsequently hydroxylated by C2’H into umbelliferone which is a pivotal intermediate in the biosynthetic pathway of more complex coumarin derivatives (Vialart et al., 2012). The cinnamates undergo *ortho*-hydroxylation of the aromatic ring, *trans/cis* isomerization of the side-chain, and lactonization to form coumarin skeleton (Shimizu, 2014).

**FIGURE 1:**
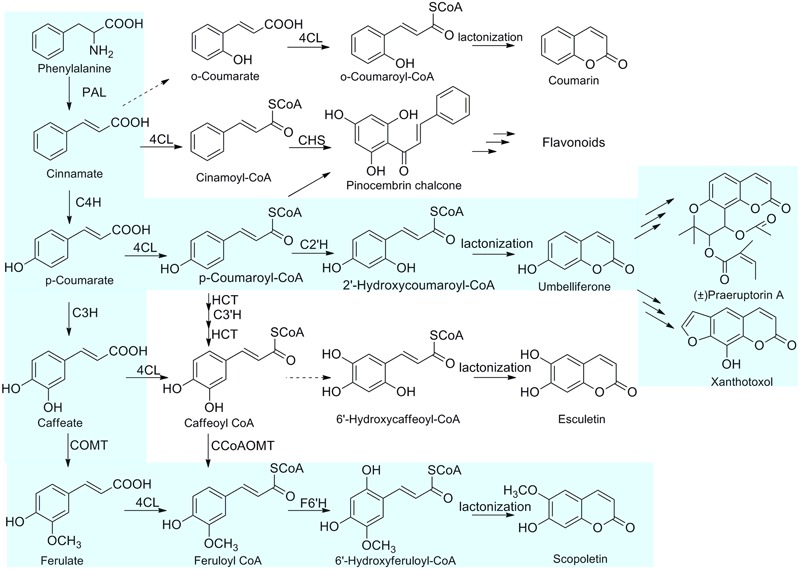
**The proposed biosynthetic pathways of coumarins in *P. praeruptorum*.** The primary for the biosynthesis of coumarins in *P. praeruptorum* are highlighted in light blue. PAL, phenylalanine ammonia-lyase; C4H, cinnamic acid 4-hydroxylase; C3H, 4-coumaric acid 3-hydroxylase; COMT, caffeic acid *O*-methyltransferase; 4CL, 4-coumarate: CoA ligase; C3′H, *p*-coumaroylshikimate/quinate 3-hydroxylase; F6′H, feruloyl CoA 6′-hydroxylase; CHS, chalcone synthase; C2′H, *p*-coumaroyl CoA 2′-hydroxylase.

4CL plays an essential role in the biosynthesis of coumarin skeletons. It could convert different hydroxycinnamyl substrates into Coenzyme A (CoA)-linked intermediates (**Figure 1**). In view of the 4CL committed roles in biosynthetic pathway of phenylpropanoid-derived metabolites, there have been a lot of reports about the cloning and identification of 4CLs from various plants since the early 1980s (Allina et al., 1998; Ehlting et al., 1999; Silber et al., 2008; Gui et al., 2011). The first 4CL gene was cloned from parsley (Douglas et al., 1987). In addition, 4CLs with higher activity have been applied to the optimization of heterologous pathways for production of bioactive compounds. Multiple studies showed that 4CL genes were involved in metabolic engineering of the complete pathway leading to heterologous biosynthesis of various compounds with health-promoting activities, such as resveratrol, naringenin and curcuminoid (Trantas et al., 2009; Wang et al., 2011; Lin et al., 2013; Rodrigues et al., 2015).

Most 4CL proteins found in higher plants have multiple isoforms, which belong to a 4CL gene family. 4CL isoforms commonly have distinct catalytic properties and expression profiles, and regulate CoA ligation fluxes in specific plant tissue. For instance, in *Arabidopsis*, At4CL1 and At4CL2 are mainly expressed in lignifying cells and are involved in lignin formation; whereas At4CL3 is expressed in a broad range of cell types and participates in the flavonoids biosynthetic pathway; At4CL4 is hardly detectable and makes a modest contribution to lignin biosynthesis (Ehlting et al., 1999; Hamberger and Hahlbrock, 2004; Li et al., 2015). A total of 17 gene models for 4CL were identified from the genome of *Populus trichocarpa*. Ptr4CL3 and Ptr4CL5 have abundant transcripts in differentiating xylem. Ptr4CL3 regulates *p*-coumaric acid and caffeic acid CoA ligation paths with similar efficiencies, whereas Ptr4CL5 primarily regulates the caffeic acid path. Moreover, Ptr4CL5 and caffeic acid coordinately modulate the CoA ligation flux for monolignol biosynthesis. Ptr4CL4 may participate in the biosynthesis of flavonoids and other soluble phenolics (Hu et al., 1998; Shi et al., 2010; Chen et al., 2013). Five 4CL isoforms from rice show different catalytic turnover rates and substrate specificities. Os4CL2 is specifically expressed in the anther and is potentially involved in flavonoid formation. Suppression of Os4CL3 expression results in lower lignin content and, thus, Os4CL3 may be involved in lignin biosynthesis (Gui et al., 2011; Sun et al., 2013).

According to genomic studies, the 4CL gene family commonly includes multiple members, with additional 4CL-like genes (Hamberger et al., 2007; De Azevedo Souza et al., 2008). 4CL-like genes belong to adenylate-forming enzymes, a large family of proteins in plants, and they show high similarity to true 4CL genes with a conserved AMP binding domain (Box I) and Box II domain (GEICIRG). Different from true 4CLs, 4CL-like enzymes commonly lack activity toward hydroxycinnamate substrates (*p*-coumaric, caffeic, ferulic, and sinapic acids) and most of them have unknown functions. However, several 4CL-like genes have been observed to have specific biochemical functions. For instance, one 4CL-like protein (At1g20510) was observed to have cyclopentane-1-octanoic acid (OPC-8) CoA ligase activity and this activity is an essential step in jasmonate biosynthesis (Kienow et al., 2008).

*Peucedanum praeruptorum*, the species of the family Apiaceae, is commonly used as a traditional Chinese medicine and its roots are rich in bioactive coumarins (Kong et al., 1996). Elucidation of the biosynthetic mechanisms of coumarins will contribute to improving the medicinal properties of *P. praeruptorum*. However, little is known about the biosynthetic enzymes of coumarins in *P. praeruptorum*. To unveil the routes for biosynthesis of the coumarins, we previously examined the transcriptome of *P. praeruptorum* and used transcriptomics in combination with metabolomics to discover candidate genes that participated in the biosynthesis and transport of coumarins (Zhao et al., 2015). Subsequently, we biochemically characterized the BMT responsible for the formation of coumarins in *P. praeruptorum* (Zhao et al., 2016). Here, we describe the cloning of three novel full-length cDNAs encoding 4CL and enzymatic characterization of Pp4CL1 involved in the biosynthesis of coumarins in *P. praeruptorum* using *Escherichia coli* recombinant proteins. Pp4CL1 catalyzes *p*-coumaric, ferulic, caffeic, cinnamic, isoferulic, and *o*-coumaric acids into corresponding hydroxycinnamate CoA thioesters and exhibits higher activity toward *p*-coumaric and ferulic acids. Consequently, Pp4CL1 may primarily regulate the *p*-coumaric acid and ferulic acid paths to achieve biosynthesis of coumarins, mainly umbelliferone, scopoletin as well as corresponding derivatives (**Figure 1**). Based on homology modeling and site-directed mutagenesis, residues essential for the enzymatic activity of Pp4CL1 are identified. Furthermore, Pp4CL7 and Pp4CL10 are identified as 4CL-like genes with unknown functions. Tissue-specific and stress-induced expression patterns of three genes were also investigated. This study provides a good candidate enzyme which adequately converts the metabolic intermediates into the desired products in metabolic engineering.

## Experimental Procedures

### Plant Materials

*Peucedanum praeruptorum* seeds were sown in pots containing soil and grown in a green house at 25°C, with 16 h daylight. These plants were used for further experiments when they were one or 6 months old.

### Isolation of 4CL Genes

Six-month-old *P. praeruptorum* plants were used for total RNA isolation. To obtain desired missing sequences, 5′ and 3′ RACE transcripts were generated using the SMARTer^TM^ RACE DNA Amplification Kit (Clontech Laboratories, Inc., Mountain View, CA, USA). The gene-specific primers used in this experiment are listed in Supplementary Table S1. The full-length cDNAs were verified by reamplification of the open reading frame (ORF) using forward primer and reverse primer (Supplementary Table S1). The PCR products of expected size were excised from the goldview-stained 1% (w/v) agarose gels, purified using the SanPrep Column DNA Gel Extraction Kit and ligated into the pMD19-T (Takara) vector for sequencing. The coding regions of genes were then deposited into the National Center for Biotechnology Information (NCBI) with the accession numbers of KX254614 (Pp4CL1), KX254613 (Pp4CL7), KX254612 (Pp4CL10).

### Construction of a Phylogenetic Tree

Amino acid sequences were aligned by ClustalX program. Alignment results were analyzed to construct a phylogenetic tree using the neighbor-joining method (Tamura et al., 2011). Statistical robustness was ensured by the bootstrap test with 1,000 replicates. Default parameters were used for all applications. Accession numbers of the sequences used in this study are listed in Supplementary Table S2.

### Expression and Purification of Recombinant Pp4CL1

The coding region of Pp4CL1 was PCR-amplied using the forward and reverse primers 5′-ATGGGAGATTATGTAGCACCCAA-3′ and 5′-CATCCGGTGATCTTCCCAAATAA-3′, respectively and cloned into a pMD19-T (TaKaRa) vector for sequencing. Then the gene was subcloned into the *Nde* I-*EcoR* I site of the expression vector pET-28a containing a cleavable N-terminal His_6_-tag. After verification by sequence analysis, the recombinant plasmid was transformed into *E. coli* BL21 (DE3) competent cells. Bacterial strain containing recombinant plasmid was grown in Luria-Bertani medium with 50 mg/L-kanamycin at 37°C. The expression of Pp4CL1 was induced with a final concentration of 0.4 mM isopropyl β-D-1-thiogalactopyranoside (IPTG) when the cultures’ density reached *A*_600_ = 0.5. After 8 h incubation on a rotary shaker (100 rpm) at 18°C, the *E. coli* cells were harvested by centrifugation and resuspended in buffer containing 50 mM NaH_2_PO_4_, 300 mM NaCl, pH 8.0. The cells were sonicated for 20 min under cooling conditions. The lysates were centrifuged for 30 min (12,000 rpm, 4°C), and the recombinant protein was purified by Ni-NTA affinity column using FPLC (ÄKTA, GE Healthcare Bio-Sciences) at 0.8 ml/min flow rate. Columns were washed by 50 mL 30 mM imidazole in a buffer containing 50 mM NaH_2_PO_4_ and 300 mM NaCl, pH 8.0. Pp4CL1 protein was eluted using a solution containing 50 mM NaH_2_PO_4_, 300 mM NaCl, and 250 mM imidazole, pH 8.0. Eluted fractions (3 mL each) with the highest protein levels were desalted on centrifugal filter devices (Amicon Ultra-4) into the buffer containing 20 mM NaH_2_PO_4_, pH 8.0. Desalted enzymes were stored at -80°C until use. The purification efficiency was monitored by SDS-PAGE (Supplementary Figure S1) and the protein concentration was determined by the Bradford method (Bradford, 1976).

### Enzymatic Activity Assays

To examine the biochemical properties of Pp4CLs, enzyme activity was assayed following previous methods with minor modifications (Knobloch and Hahlbrock, 1975). Briefly, the reaction mixture (200 μl) contained 50 mM NaH_2_PO_4_, 300 mM NaCl, 5 mM ATP, 5 mM MgC1_2_, 3 μg of purified recombinant 4CLs protein and 0.3 mM substrates (*p*-coumaric acid, caffeic acid, ferulic acid, and *trans*-cinnamic acid, etc.), pH 8.0. The CoA (0.3 mM) was added to the mixture to initiate the reaction. After being incubated for 15 min at 37°C, the mixture was boiled for 10 min and centrifuged (10 min, 12000 rpm) to remove the protein. Blanks contained 0.3 mM substrate, 5 mM ATP, 5 mM Mg^2+^, 50 mM NaH_2_PO_4_, 300 mM NaCl and 3 μg of purified protein. UV absorptions were recorded continuously for 15 min at an interval of 60 s, using the blanks as background. Formations of the corresponding CoA esters were determined by monitoring the increase in absorption maximum at wavelengths 310 nm for cinnamoyl-, 333 nm for *p*-coumaroyl-, 333 nm for *o*-coumaroyl-, 346 nm for caffeoyl-, 346 nm for isoferuloyl- and 346 nm for feruloyl-CoA esters (Lee et al., 1997). The optimum temperature and pH of Pp4CL1 enzyme were analyzed, using 4-coumaric acid as substrate.

### HPLC/DAD-Q-TOF-MS/MS Analysis and Product Determination

The reaction products were dried under nitrogen gas and then were dissolved in methanol for HPLC analysis. The processed samples (10 μl) were subjected to an Agilent 1290 LC System which was coupled to a multiple-wavelength diode array detector and equipped with a ZORBAX SB-C18 column (4.6 × 250 mm, 5 μm) for analysis. Separation column was maintained at 30°C with a flow rate of 1.0 mL/min. The mobile phase consisted of solvents A (0.1% ammonium acetate in double distilled water) and B (methanol). Gradient elution was done for 20-min and the conditions were as follows: 0–5 min, 5–10% B linear; 5–15 min, 10–60% B linear; 15–20 min, 60–90% B linear. Elution of compounds was monitored at 333 nm (*p*-coumaroyl-CoA and *o*-coumaroyl-CoA), 346 nm (caffeoyl-CoA, feruloyl-CoA, and isoferuloyl-CoA) and 310 nm (*trans*-cinnamoyl-CoA).

All reaction products were identified by LC-MS, using the above conditions. The HPLC system was coupled to Q-TOF MS spectrometer (Agilent Technologies, Santa Clara, CA, USA), equipped with an electrospray source in positive ion mode. The conditions of ESI source were as follows: drying gas (N_2_) flow rate, 8.0 mL/min; nebulizer, 241 kPa (35 psig); fragmentor voltage, 150 V; collision energy, 30 eV; octopole radio frequency, 250 V and skimmer voltage, 60 V. In addition, the capillary voltage was set at 4.0 kV and the desolvation gas temperature was 325°C.

### Gene Expression Analysis and Induction of Pp4CLs Expression by Elicitors

For methyl jasmonate (MeJA) elicitation, the roots of plants were treated with MeJA (Sigma–Aldrich, 95% purity) at a final concentration of 0.2 mM in 10% ethanol. The control was treated with the same amount of ethanol. All samples were harvested for analysis at 3, 6, 9, 12, and 24 h after elicitation. Meanwhile, the plants were also treated with PEG, NaCl, CuSO_4_, heat and cold for a certain time. The above samples were ground in liquid nitrogen with a mortar and pestle and then stored at -80°C for RNA extraction.

Total RNA were extracted from *P. praeruptorum* roots, stems, leaves, seedlings in turn, using Trizol reagent following the manufacturer’s instructions (Invitrogen). The quantity and quality of RNA were determined using a Spectramax plus384 enzyme-labeling instrument (Molecular Devices, Sunnyvale, CA, USA) and 1% agarose gels. Total RNA were treated with RNase-free DNase I to remove DNA contamination and were reverse transcribed into cDNA using Vazyme’s HisScript QRT SuperMix. Gene expression was assayed by quantitative real-time PCR (qPCR) using the cDNA equivalent of total RNA. Gene-specific primers were designed for PCR amplification (Supplementary Table S1). qPCR was performed on a Roche Light Cycler 480 instrument using a 96 well plate by Vazyme’s SYBR Green PCR Master Mix. The PCR conditions for the amplification of 4CLs transcripts were as follows: 5 min of denaturation at 95°C, 40 cycles of 95°C for 30 s, 60°C for 30 s, followed by one cycle of 95°C for 15 s, 60°C for 60 s, and 95°C for 15 s. The gene expression values were calculated by 2^-ΔΔCt^ method (Schmittgen, 2006) and were normalized using GAPDH gene as a reference. At least three technical repetitions were performed.

### Homology Modeling and Site-Directed Mutagenesis

The crystal structure of *Populus tomentosa* 4CL1 (Hu et al., 2010) was used to conduct a structure model for Pp4CL1. Based on the homology modeling and sequence alignment, some essential residues were selected for site-directed mutagenesis to investigate the key amino acid residues which made an effect on enzymatic specificity and activity. The Pp4CL1 mutants were generated using overlap PCR method and the primers used in this experiment are shown in Supplementary Table S1. Mutated genes were confirmed by sequencing and then transferred into *E. coli* BL21 (DE3). Purification and activity assays of mutant recombinant proteins were conducted using the same protocol with wild-type Pp4CL1. The substrates used in enzyme assays were *p*-coumaric, caffeic, ferulic, and isoferulic acids. The reaction products were analyzed by HPLC.

### GFP Fusion Construct to Analyze the Subcellular Localization of Pp4CLs

Construction of GFP fusion with the N terminal region of Pp4CLs and the transient expression in *Arabidopsis* protoplasts were conducted according to previously described protocol (Zhao et al., 2016). For amplification of Pp4CLs (Pp4CL1 and Pp4CL10), primers were designed and listed in Supplementary Table S1. Plasmid pCAMBIA1302-GFP with the cauliflower mosaic virus (CaMV) 35S promoter was used for the generation of GFP fusion constructs. The PCR fragments of two genes were subcloned into the pCAMBIA1302 vector in frame with the GFP. The ORF of PEX7 (*Arabidopsis thaliana*) was amplified by PCR using PTS1-Per-mcherry-F: CGCTTCTAGAATGCCGGTGTTCAAAGCTCC (*Xba I*) and PTS1-Per-mcherry-R: AATCCCCGGGACTGGCTCTAGGATCCATCCC (*Sma I*). The PCR fragment was cloned into P16ΔS:sXVE:mCherryC vector (Schlücking et al., 2013). *Arabidopsis* protoplasts were prepared and then transformed with the constructed pCAMBIA1302-4CLs plasmids and pCAMBIA1302 empty vector, respectively, using polyethylene glycol (PEG)-mediated transient gene expression (Wu et al., 2009). Meanwhile, *Arabidopsis* protoplasts were also co-transformed with pCAMBIA1302-Pp4CL1 plasmid and the constructed plasmid P16ΔS:sXVE:mCherryC-PEX7 (peroxisomal marker). Transient expression of GFP fusion proteins was observed 16 h after transformation by a laser scanning confocal microscope (LSM 780; Carl Zeiss, Jena, Germany), equipped with 20 × 0.8 Plan-Apochromat, 40 × 1.2 W C-Apochromat or 63 × /1.4 Oil Plan-Apochromat in multitrack channel mode.

## Results

### Identification of cDNAs Encoding 4CL in *P. praeruptorum*

A total of 17 cDNA fragments encoding putative CoA ligases (annotated as 4CL or 4CL-like) were identified in previous experiment (Zhao et al., 2015). Here, we investigated the expression profiles of these putative 4CL genes and three 4CL genes with relatively high expression levels were selected for further study. The full-length cDNA of genes were identified by 5′ and 3′ rapid amplification of cDNA ends (RACE). The three genes were named as Pp4CL1, Pp4CL7 and Pp4CL10. Pp4CL1 shared high nucleotide identity with 4CL1 from *Petroselinum crispum*, which, like *P. praeruptorum*, also belongs to Apiaceae family. The ORF lengths of Pp4CL1, Pp4CL7 and Pp4CL10 were 1629 bp (encoding 543 residues), 1626 bp (encoding 542 residues) and 1566 bp (encoding 522 residues), respectively. The amino acid sequences of the three genes contained highly conserved AMP-binding motif (Box I) and a Box II domain (GEICIRG domain).

### Phylogenetic Analysis and Sequence Alignment

**FIGURE 2:**
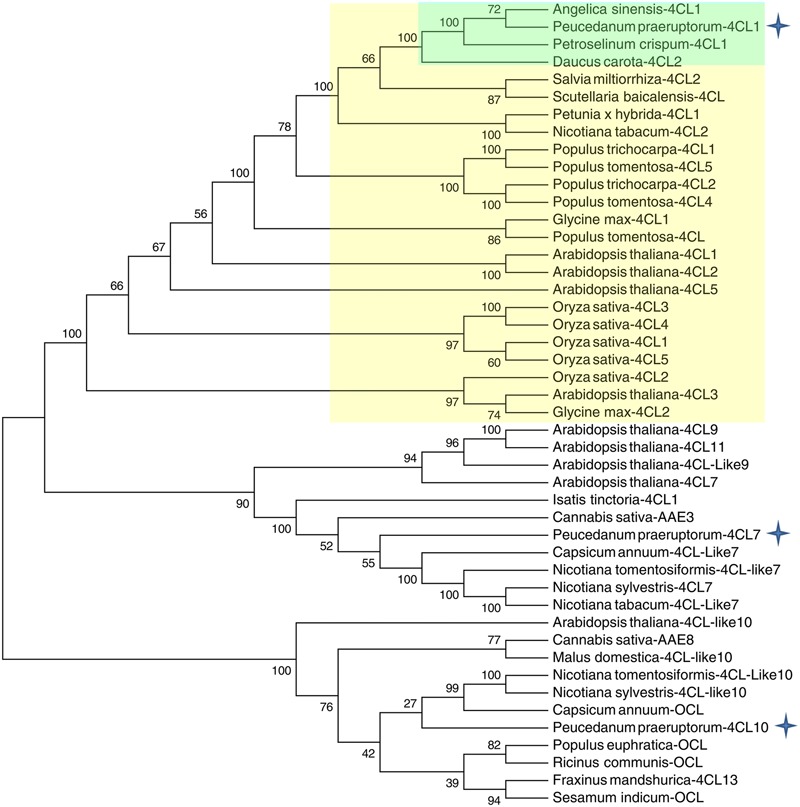
**Phylogenetic relationships between Pp4CLs and 4CLs from different plants.** The neighbor-joining tree was constructed through MEGA 5.05 with 1000 replicates bootstrap support. The bootstrap values are marked at the branch points. The accession numbers of the proteins used for the preparation of this tree are listed in Supplementary Table S2.

**FIGURE 3:**
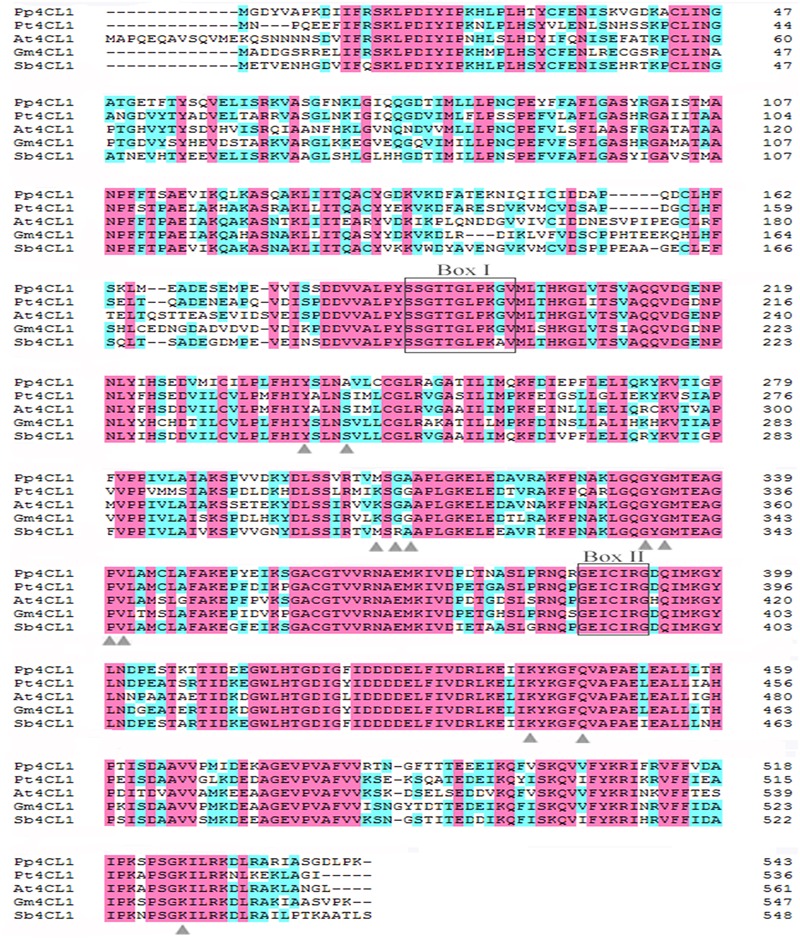
**Alignment of the amino acid sequences of Pp4CL1 and other plant 4CLs.** The amino acid sequences shown are *P. praeruptorum* 4CL1 (Pp4CL1; KX254614), *Arabidopsis thaliana* 4CL1 (At4CL1; NP_001077697), *Glycine max* 4CL1 (Gm4CL1; NP_001236418.1), *Populus tomentosa* 4CL1 (Pt4CL1; AAL02144.1), *Scutellaria baicalensis* 4CL1 (Sb4CL1; BAD90936.1). The conserved peptide motifs Box I and II are highlighted. Black letters on pink background are designated as completely conserved residues. Black letters on blue background are designated as highly conserved residues. Residues proposed to function in 4CL substrate specificity are marked with triangles.

A phylogenetic tree was constructed based on a series of 4CLs that have actual or putative activity toward *p*-coumaric acid or derivatives. Protein accession numbers and plant species used in the phylogenetic tree are shown in Supplementary Table S2. Phylogenetic analysis revealed that Pp4CL1, Pp4CL7, and Pp4CL10 belong to three different branches of the evolutionary tree (**Figure 2**). Pp4CL1 belonged to one clade in which related genes have traditional 4CL protein function. Pp4CL1 had higher homology with As4CL (*Angelica sinensis*), Pc4CL (*Petroselinum crispum*) and Dc4CL1 (*Daucus carota*), and the corresponding plants are all derived from the Umbelliferae family. Whereas, Pp4CL7 and Pp4CL10 were classified as members of other clades in which related proteins have not been demonstrated to possess 4CL activity. The sequence alignment was performed using related 4CL proteins with high sequence similarity. Pp4CL1 contains a highly conserved AMP-binding motif (Box I) and a Box II domain (GEICIRG domain). Some residues essential for the enzyme activity have been marked (Hu et al., 2010). The Ser-240 in Pt4CL1, highly conserved in 4CLs, was replaced by Ala-243 and key residues Lys-303 and Gly-306 in Pt4CL1 were also substituted by Met-306 and Ala-309 here, respectively (**Figure 3**).

### Elicitor-Induced 4CLs Transcription and Expression Profile in *P. praeruptorum*

**FIGURE 4:**
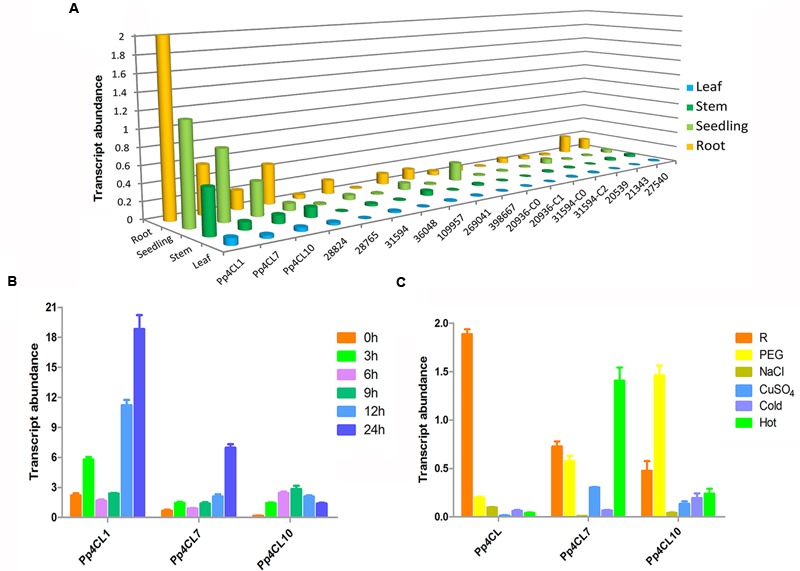
**Expression profiles of Pp4CLs. (A)** Tissue-specific expression of 17 putative 4CL genes. **(B)** Transcript abundance of three 4CL genes in roots at 0, 3, 6, 9, 12, and 24 h after MeJA treatment. **(C)** Expression level of three genes in *P. praeruptorum* with various treatments. “R” represents roots without any treatment. Values are means ± SE (*n* = 3).

The transcript levels of three 4CL genes in various tissues of *P. praeruptorum* were quantified by qPCR using gene-specific primers (Supplementary Table S1). The three 4CL genes showed different expression patterns in different tissues. The results showed that expression levels of Pp4CL1, Pp4CL7, and Pp4CL10 in roots were higher than those of leaves and stems. Meanwhile, Pp4CL1 had the highest expression level among all putative 4CL genes (**Figure 4A**). In addition, transcript levels of Pp4CL1 increased in mature roots compared with those in seedlings, which correlates to the accumulation of bioactive coumarins in roots. MeJA had strong effects on the expression levels of 4CLs in roots. Upon MeJA treatment, the transcript levels of three genes were significantly up-regulated, with the increasing duration of interaction (**Figure 4B**). Transcript levels of Pp4CL1 and Pp4CL7 in roots reached the maximum after 24 h whereas the expression level of Pp4CL10 was clearly enhanced at 9 h and then declined (**Figure 4B**). Subsequently, we also investigated the expression patterns of three genes in response to abiotic stresses such as PEG, NaCl, CuSO_4_, heat and cold treatments. Upon the above, the expression level of Pp4CL1 significantly decreased. However, transcript abundance of Pp4CL7 was up-regulated under heat treatment, and the expression level of Pp4CL10 was clearly enhanced under PEG treatment (**Figure 4C**).

### Functional Analysis and Characterization of Pp4CLs

**FIGURE 5:**
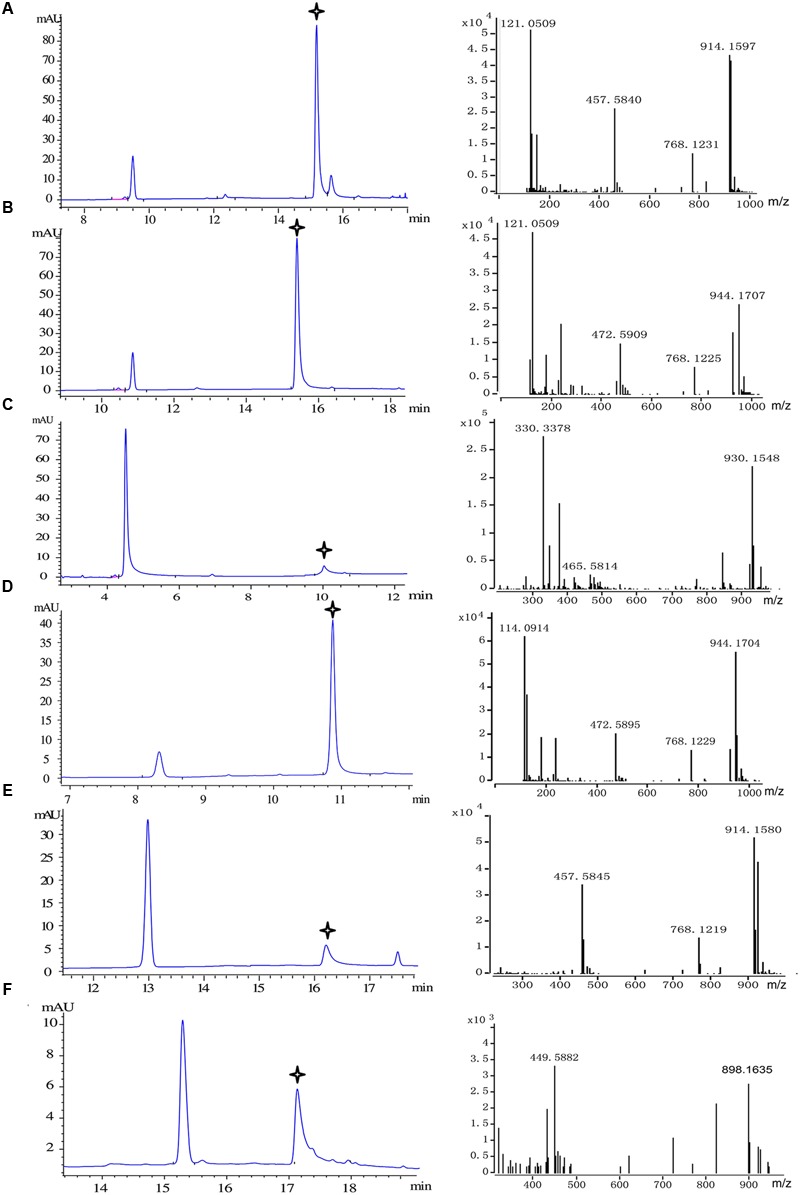
**Substrate specificity of recombinant Pp4CL1.** HPLC analysis and Q-TOF-MS identification of reaction products generated by recombinant Pp4CL1 protein. **(A)** *p*-Coumaric acid as substrate. **(B)** Ferulic acid as substrate. **(C)** Caffeic acid as substrate. **(D)** Isoferulic acid as substrate. **(E)** *o*-Coumaric acid as substrate. **(F)** Cinnamic acid as substrate.

For purposes of determining whether Pp4CL1, Pp4CL7, and Pp4CL10 had the CoA ligase function, the coding regions of these cDNAs were cloned and expressed in *E. coli* BL21 (DE3). The activity of purified recombinant proteins toward several substrates, including *p*-coumaric, *o*-coumaric, ferulic, caffeic, cinnamic, isoferulic, and sinapic acids were tested (**Figure 5**). The results revealed that Pp4CL1 had distinct CoA ligation activity toward the above substrates except sinapic acid. Furthermore, Pp4CL1 exhibited higher activities toward *p*-coumaric and ferulic acids than other substrates. Pp4CL7 and Pp4CL10 had no activity toward hydroxycinnamic acid compounds (data not shown), indicating that they were not CoA ligases involved in the biosynthesis of coumarins, which was in agreement with the analysis of the phylogenetic tree. Therefore, Pp4CL7 and Pp4CL10 were classified as 4CL-like genes, which were annotated as being closely related to true 4CLs but of unknown biochemical function. To further confirm whether the two genes could function as fatty acid CoA ligases, a series of fatty acids were tested as substrates using recombinant Pp4CL7 and Pp4CL10 proteins. The results showed that neither of them had activity toward the fatty acids (data not shown).

We then focused on the specific properties of the Pp4CL1 enzyme. Pp4CL1 displayed extensive catalytic activities for hydroxycinnamate derivatives by converting them into the corresponding CoA esters. All CoA ligation reactions were monitored by HPLC and the identities of CoA thioester products were established by time-of-flight liquid chromatography-mass spectrometry (LC/TOF-MS) in positive ion mode. The six corresponding product peaks were detected in accordance with analysis results of HPLC profiles (**Figure 5**) and the reaction products were identified by high resolution mass spectrometry (HRMS). Dominant ions of *p*-coumaroyl-CoA were singly and doubly charged pseudo molecular ion [M + H]^+^ at *m/z* 914.1597 and [M + 2H]^2+^ at *m/z* 457.5840, respectively (**Figure 5A**). Dominant ions of feruloyl-CoA were *m/z* 944.1707 and *m/z* 472.5909 (**Figure 5B**). Dominant ions of all products identified by HRMS are also listed in Supplementary Table S3. In addition, the optimum pH of Pp4CL1 was approximately 6.5 (Supplementary Figure S2) and its optimum temperature was 37°C (Supplementary Figure S3).

### Subcellular Localization of Pp4CLs

**FIGURE 6:**
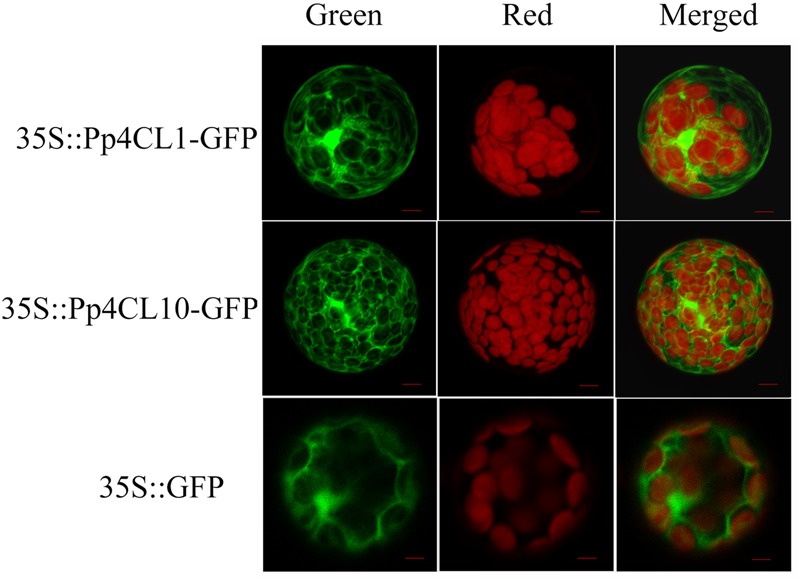
**Subcellular localizations of *P. praeruptorum* 4CL1 and 4CL10.** “Green” panels show GFP fluorescence, and chloroplast autofluorescence is shown in “Red” panels. “Merged” panels represent combined fluorescence from GFP and chloroplasts. *Arabidopsis* protoplasts containing empty vector were used as a control, namely 35S::GFP. 35S::Pp4CL1-GFP represents protoplasts containing pCAMBIA1302-4CL1 plasmid. Protoplasts containing pCAMBIA1302-4CL10 plasmid are abbreviated as 35S:: Pp4CL10-GFP. Bars = 20 μm.

It was predicted that Pp4CL1 and Pp4CL10 are localized in the cytosol using WoLF PSORT program. To further investigate the subcellular localization of the Pp4CL1 and Pp4CL10, their ORFs were cloned into pCAMBIA1302 so that the proteins were fused with green fluorescence protein (GFP) gene under the control of the 35S promoter from CaMV. The designed constructs were transferred into protoplasts of *Arabidopsis* by PEG-mediated transformation (Wu et al., 2009). The GFP:4CL fusion proteins were expressed in *Arabidopsis* protoplast. *Arabidopsis* protoplast containing the empty vector was used as a control. The green fluorescence was present throughout the cytoplasm in *Arabidopsis* protoplasts containing Pp4CL1-GFP. There was no overlap between GFP and the red autofluorescence of chlorophyll (**Figure 6**), showing a typical fluorescence pattern of cytosol localization. A similar pattern was observed for the Pp4CL10-GFP fusion protein and the GFP control (**Figure 6**). The results verified that the Pp4CL1 and Pp4CL10 proteins were localized to the cytosol of plant cells. Furthermore, *Arabidopsis* protoplasts were co-transformed with Pp4CL1 and PEX7 (*Arabidopsis*). PEX7 is known to localize in peroxisomes (Singh et al., 2009). There was no overlap between the GFP and the red fluorescent of PEX7, providing evidence that the Pp4CL1 protein is not localized in peroxisomes (Supplementary Figures S4A–D).

### Site-Directed Mutagenesis of Pp4CL1

**FIGURE 7:**
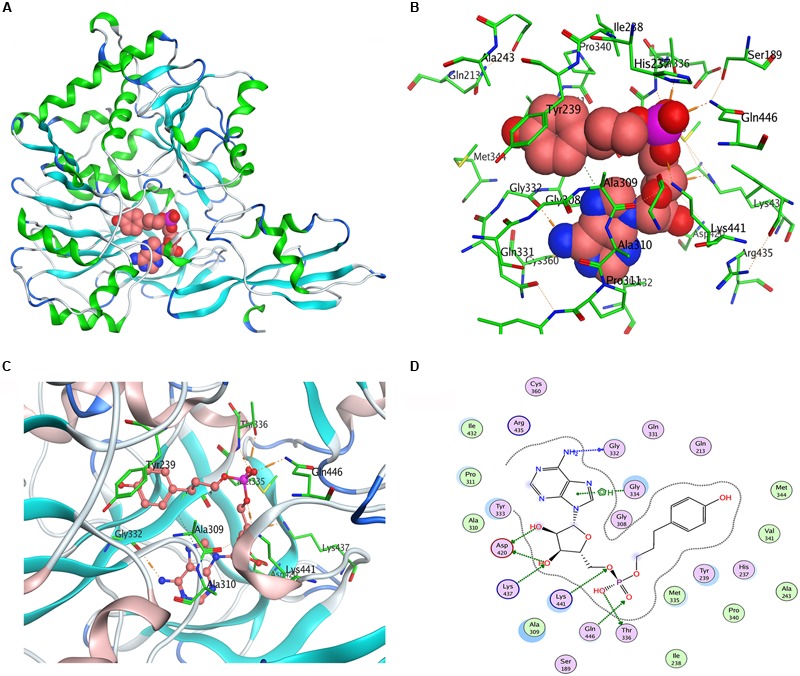
**Homology modeling and docking of Pp4CL1 with APP. (A)** Three-dimensional model of Pp4CL1-APP complex. **(B)** The hydroxycinnamate binding pocket of Pp4CL1. **(C)** Stereoview of the Pp4CL1-APP interaction. **(D)** Two-dimensional model of Pp4CL1-APP complex. APP, adenosine 50-(3-(4-hydroxyphenyl) propyl) phosphate.

**FIGURE 8:**
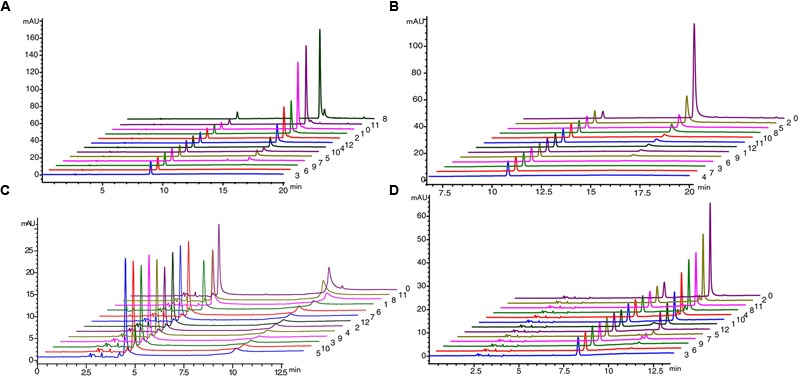
**HPLC profile analysis of reaction products generated by mutations of Pp4CL1 (A)** HPLC profile of mutations using *p*-coumaric acid as substrate. **(B)** HPLC profile of mutations using ferulic acid as substrate. **(C)** HPLC profile of mutations using caffeic acid as substrate. **(D)** HPLC profile of mutations using isoferulic acid as substrate. Numbers 0-12 represent: wild-type Pp4CL1 (0), Y239A (1), Y239F (2), Y239W (3), A243S (4), M306K (5), M306A (6), G308A (7), A309G (8), G334A (9), K441A (10), Q446A (11) and K526A (12).

We used *Populus tomentosa* 4CL (PDB ID: 3NI2) as a template to build a meaningful three-dimensional model of Pp4CL1-APP complex (**Figure 7A**). Key amino acids in the hydroxycinnamate binding pocket were Tyr239, Ala243, Gly308, Ala309, Gly332, Gly334, Thr336, Lys437, Lys441, Gln446 (**Figures 7B,C**). Some amino acids, such as Lys437 and Gln446, form hydrogen bonds with the substrate (**Figure 7D**). Based on homology modeling and multiple sequence alignments, some mutants, such as Y239A, Y239F, Y239W, A243S, M306K, M306A, G308A, A309G, G334A, K441A, Q446A, and K526A, were generated. We tested the enzymatic activities of mutants toward four substrates (*p*-coumaric, ferulic, caffeic, and isoferulic acids). The results of HPLC analysis are shown in **Figure 8** and the data are listed in Supplementary Table S4. Mutations of Y239W and G334A almost abolished Pp4CL1 activity, probably because Trp and Ala amino acids decrease the affinity of Pp4CL1 for its substrates. According to the sequence alignment results, the three essential residues of Pp4CL1, namely Ala-243, Met-306 and Ala-309, were different from other 4CLs. The current research focused on mutations of M306A, M306K, A309G and A243S. The results showed that the M306A mutation completely abolished Pp4CL1 activities toward *p*-coumaric and ferulic acids and diminished its activities toward caffeic and isoferulic acids (**Figure 8**). However, the mutation of M306K only decreased Pp4CL1 activities. As part of the binding pocket, the amino acid Ala-309 was mutated into Gly and the mutation increased Pp4CL1 activity toward *p*-coumaric acid, but diminished its activities toward the other three substrates. Ser-240 in Pt4CL was highly conserved in 4CL genes. However, the corresponding amino acid in Pp4CL1 was Ala. The mutation of A243S completely abolished Pp4CL1 activity toward ferulic acid. In addition to the above mutations, there were also other essential mutations in this study and most mutations reduced activities of Pp4CL1 toward substrates to different degrees (**Figure 8**).

## Discussion

The 4CL plays an essential role in the phenylpropanoid pathway because it catalyzes the formation of hydroxycinnamoyl-CoA thioesters, precursors for the synthesis of lignins, monolignols, flavonoids, stilbenes, coumarins, and other phenylpropanoids. Previous studies on 4CL genes focused on their key roles in biosynthetic pathway of lignins, monolignols and flavonoids (Yang et al., 2011; Saballos et al., 2012; Chen et al., 2013; Sun et al., 2013), but little is known about the properties of 4CL genes participating in the biosynthesis of coumarins. The roots of *P. praeruptorum* are commonly used as traditional Chinese drug and its main active compounds are coumarins. In this study, three 4CL genes were first isolated from *P. praeruptorum* and were defined as Pp4CL1 (bona fide 4CL), Pp4CL7 and Pp4CL10, respectively. Phylogenetic analysis suggested that they may have different functions. Pp4CL1 was enzymatically characterized and had broad substrate specificity, catalyzing *p*-coumaric, *o*-coumaric, ferulic, caffeic, cinnamic and isoferulic acids into corresponding hydroxycinnamate CoA thioesters. Moreover, Pp4CL1 protein had higher activity toward *p*-coumaric and ferulic acids than any other substrates. Because of the structural similarity of isoferulic acid and ferulic acid, Pp4CL1 also had better activity toward isoferulic acid. Pp4CL1 represents a bona fide 4CL and shows different catalytic function from the other 4CL enzymes (Ehlting et al., 1999; Hu et al., 2010; Chen et al., 2013; Gao et al., 2015). Pp4CL1 mainly regulates the biosynthesis of coumarins. However, Pp4CL7 and Pp4CL10 were identified as 4CL-like genes that encode proteins that share structural similarities with bona fide 4CLs, such as conserved Box I and II domains and substrate binding domains. They also have close phylogenetic relationships with true 4CLs (Schneider et al., 2003; De Azevedo Souza et al., 2008). To date, most 4CL-like genes possess unknown specific biochemical functions and only a few of them have been identified (Koo et al., 2006; Kienow et al., 2008).

The transcript abundances of the three genes were investigated in different tissues and under different treatments using qPCR. The three genes had higher transcript abundances in roots than in stems and leaves, which is in agreement with a higher content of coumarins in roots (Zhao et al., 2015). Among all putative 4CLs, Pp4CL1 had the highest expression level. The expression level of Pp4CL1 was higher in mature plants than in seedlings. These results suggest that Pp4CL1 is more likely to be involved in the biosynthesis of coumarins in *P. praeruptorum*, and that Pp4CL1 plays a crucial role in this biosynthetic pathway. The higher activity of Pp4CL1 protein toward 4-coumarate and ferulate may explain the accumulation of umbelliferone and scopoletin in *P. praeruptorum* (**Figure 1**). We also conducted experiments about subcellular localizations of Pp4CLs for the first time. The results showed that both Pp4CL1 and Pp4CL10 proteins were localized in the cytosol (**Figure 6**). Pp4CL1 protein has the same localization as Ph4CL1 (*Petunia hybrid*) (Klempien et al., 2012). According to previous reports, 4CL protein is mainly localized in the cytosol or peroxisomes (Klempien et al., 2012; Gaid et al., 2012). In this study, pCAMBIA1302-Pp4CL1 plasmid and the peroxisomal marker (PEX7 gene) were co-transformed into *Arabidopsis* protoplasts. The result showed that Pp4CL1 protein was localized in the cytosol and not peroxisomes (Supplementary Figure S4). Pp4CL7 and Pp4CL10 have similar expression profiles with Pp4CL1 (**Figure 4A**). Pp4CL10 has the same subcellular localization with Pp4CL1 (**Figure 6**). However, Pp4CL7 and Pp4CL10 encode enzymes without traditional 4CL activity and so it is necessary to conduct more experiments to reveal their specific functions in the future.

Here we focused on studying enzymatic mechanisms of Pp4CL1. To investigate the important residues which affect Pp4CL1 enzyme activity and substrate specificity, a series of mutations were generated based on homology modeling (**Figure 7**) and sequence alignment (**Figure 3**). The mutations of A243S, M306A, M306K and A309G have different effects on Pp4CL1 activity. The residue Ser-240 in Pt4CL1, highly conserved in 4CLs, is substituted by residue Ala-243 here (**Figure 3**). Mutation of A243S largely diminished Pp4CL1 activities toward substrates, and completely abolished its activity toward ferulic acid, probably because Ser residue damages hydrophobic interaction and may affect tertiary structure of protein. The effect of this mutation is consistent with previous research (Gao et al., 2015). The mutation of M306A resulted in complete loss of Pp4CL1 activities toward *p*-coumaric and ferulic acids, and M306K mutant decreased activity of Pp4CL1. It means that the residue Ala-306 may destroy hydrophobic interaction and affect tertiary structure of protein. But residue Lys may form a salt bond in the protein and affect the secondary structure. When amino acid Ala-309 was replaced with Gly, the activity of the protein toward *p*-coumaric acid was enhanced, but its activity toward other substrates was reduced. The residue Ala-309 is located in the binding pocket and has a complex interaction with hydroxycinnamate.

Based on homology modeling, the residue Tyr-239 is located in the hydroxycinnamate binding pocket (**Figure 7B**). All mutations of Y239A, Y239W, and Y239F decreased Pp4CL1 activity. We propose that the residue Tyr interacts with hydroxycinnamate by hydrogen bond and other molecular forces. The Y239A mutation may weaken hydrogen bonds between Tyr-239 and hydroxycinnamate, and reduce the activity of Pp4CL1. The mutation of Y239W may result in steric hindrance, weaken affinity, and thus abolished enzymatic activity toward *p*-coumaric, ferulic, and isoferulic acids. However, the activity toward caffeic acid was still maintained, probably because the two hydroxyls of caffeic acid resulted in relatively strong hydrogen bonds with the protein. Y239F mutation decreased Pp4CL1 activity probably through disrupting the hydrogen bond interaction. Tyr-239 also forms a hydrogen bond in the protein. The mutations of Y239A, Y239W and Y239F may damage a hydrogen bond in the protein and form weaker hydrophobic interactions, and these mutations may affect secondary structure of Pp4CL1 protein. The mutation G334A totally abolished Pp4CL1 activity toward substrates and it is probably because amino acid Ala-334 reduces the size of the substrate binding pocket. The mutations of catalytic residues, such as K441A, Q446A, and K526A, did not completely abolish, but did diminish, Pp4CL1 activity. Consequently, we propose that these residues may not be strictly conserved but may be important for the enzymatic activity of Pp4CL1. Based on homology modeling, the residue Gln-446 forms a hydrogen bond with the phosphate group of the substrate. Lys-441, Gln-446, and Thr-336 interact with the phosphate group of the substrate (**Figure 7D**), and these interactions may be essential for Pp4CL1 activity.

In summary, this is the first report on the functional characterization of Pp4CL1, an enzyme involved in the biosynthesis of coumarins in *P. praeruptorum*. Pp4CL1 has broad substrate specificity for hydroxycinnamates, and possesses higher activity toward *p*-coumaric and ferulic acids than other substrates. Consequently, Pp4CL1 may primarily be involved in the *p*-coumaric acid and caffeic acid pathways to achieve the biosynthesis of coumarins, mainly umbelliferone, scopoletin and their corresponding derivatives (**Figure 1**). Pp4CL1 and Pp4CL10 proteins were localized in the cytosol. In addition, some key amino acid residues affecting substrate binding and catalytic activities were identified by site-directed mutagenesis. This study provides further insights in understanding the structure-function relationships of this important 4CL protein.

## Author Contributions

Conceived and designed the work: TL, YZ, JL, and LK. Performed the experiments: TL, RY, SX, and YZ. Interpret and analyzed the data: TL and CH. Wrote the paper: TL. Revised the paper critically: TL, RY, YZ, and JL. All authors read and approved the final manuscript.

## Conflict of Interest Statement

The authors declare that the research was conducted in the absence of any commercial or financial relationships that could be construed as a potential conflict of interest.
