# Supplementary material for: Cloning, Functional Characterization and Site-Directed Mutagenesis of 4-Coumarate: Coenzyme A Ligase (4CL) Involved in Coumarin Biosynthesis in Peucedanum praeruptorum Dunn
Source: Front Plant Sci. 2017 Jan 17;8:4. doi: 10.3389/fpls.2017.00004 (PMC5239791; doi:10.3389/fpls.2017.00004)
Supplement: Supplementary file 1 [file Data_Sheet_1.DOC]

***Supplementary Material***

**Cloning****，functional characterization and site-directed mutagenesis of** **4-coumarate: Coenzyme A ligase (4CL) involved in coumarin** **biosynthesis in** ***Peucedanum praeruptorum* Dunn**

Tingting Liu^1^, Ruolan Yao^1^, Yucheng Zhao^1^, Sheng Xu^2^, Chuanlong Huang^1^, Jun Luo^1*^ and Lingyi Kong^1*^

***Correspondence:**

Lingyi Kong

[cpu_lykong@126.com](mailto:cpu_lykong@126.com)

Jun Luo
[luojun1981ly@163.com](mailto:luojun1981ly@163.com)

**Legends of** **supplementary materials**

**Table S1. Primers used in this study**

**Table S2. Accession numbers and the names of species used in this study**

**Table S3.** **Dominant ions in high resolution mass spectra of hydroxycinnamate CoA thioesters in positive mode**

**Table S4. Relative activities (%) of Pp4CL1 mutants towards *p*-coumaric acid, ferulic acid, caffeic acid and isoferulic acid**

**Figure S1.** **SDS**-**PAGE analysis of recombinant His_6_**-**Pp4CL1 purified by affinity chromatography**

**Figure S2.** **Effects of various pHs on the activity of Pp4CL1**

**Figure S3. Effects of various temperatures on the activity of Pp4CL1**

**Figure S4. Subcellular localization of Pp4CL1 protein with additional peroxisomal marker (PEX7). GFP fluorescence is shown in green and chlorophyll autofluorescence is shown in blue. In (A), fluorescence of PEX7 (peroxin 7) used as a peroxisomal marker is shown in red. Merged panel shows combined fluorescence from GFP, chloroplasts, and peroxisomes. Bars = 20μm.**

**Table S1. Primers used in this study**

| Primer name | (5’ to 3’) |
| --- | --- |
| 4CL-P2 | TCGCTCAACGCGGTGTTGTGCTGTG |
| 4CL-P1 | GGCACCTCACCCGCTTTCTCATCGA |
| 4CL- NGSP2 | GCGAAAAGCCCGGTGGTGGACAA |
| 4CL- NGSP1 | TTCTCCCCGTTGGTTTCGTGGAAGA |
| *Nde* I-4CL-F | GGAATTCCCATATGGGAGATTATGTAGCAC |
| 4CL- *EcoR* I-R | GGAATTCTTTGGGAAGATCACCGGATGCTA |
| *Nde* I-4CL7-F | CATATGGAAAAATCCGGTTATGGCCG |
| 4CL7- *Not* I-R | GCGGCCGCTATTTTAGCTCGAACTT |
| *Nde* I-4CL10-F | CATATGGAAAATAAAACGCTCACT |
| 4CL10- *Not* I-R | GCGGCCGCCTAGGCTCCAAATTTCG |
| 4CL-Q-F | GACTTAGAGCAGGGGCGAC |
| 4CL-Q-R | ATATTTGTCCACCACCGGG |
| 4CL7-Q-F | AGATTGGGTCTGGTGCTGC |
| 4CL7-Q-R | GTCGAGGACCTCCCAATTG |
| 4CL10-Q-F | GTTCGGAGCTCCGGTTCTG |
| 4CL10-Q-R | ACCGCTTTCCCAACTGACC |
| 4CL-1302F | GAAGATCTTATGGGAGATTATGTAGCACCCA |
| 4CL-1302R | GACTAGTCAGGTCTACCATTTTGGGAAGATCAC |
| 4CL10-1302F | AGATCTTATGGAAAATAAAACGCTCACTGGCTTGTT |
| 4CL10-1302R | ACTAGTCAGGTCTACCATGGCTCCAAATTTCGGC |
| Y239A-1R | CACCGCGTTGAGCGAAGCGATATGAAACAAAGGCAGAA |
| Y239A-2F | TTCTGCCTTTGTTTCATATCGCTTCGCTCAACGCGGTG |
| Y239F-1R | ACCGCGTTGAGCGAAAAGATATGAAACAAAGGCAGA |
| Y239F-2F | TCTGCCTTTGTTTCATATCTTTTCGCTCAACGCGGT |
| Y239W-1R | CACCGCGTTGAGCGACCAGATATGAAACAAAGGCAGAA |
| Y239W-2F | TTCTGCCTTTGTTTCATATCTGGTCGCTCAACGCGGTG |
| A243S-1R | TCCACAGCACAACACCGAGTTGAGCGAATAGATATGAA |
| A243S-2F | TTCATATCTATTCGCTCAACTCGGTGTTGTGCTGTGGA |
| M306 K-1R | CGGAGCAGCTCCAGACTTAACCGTCCTCACCGACGACA |
| M306 K-2F | TGTCGTCGGTGAGGACGGTTAAGTCTGGAGCTGCTCCG |
| M306 A-1R | CGGAGCAGCTCCAGACGCAACCGTCCTCACCGACGACA |
| M306 A-2F | TGTCGTCGGTGAGGACGGTTGCGTCTGGAGCTGCTCCG |
| G308A-1R | CCTAACGGAGCAGCTGCAGACATAACCGTCCTCACCG |
| G308 A-2F | CGGTGAGGACGGTTATGTCTGCAGCTGCTCCGTTAGG |
| A309 G-1R | TTTCCCTAACGGAGCACCTCCAGACATAACCGTCCTCA |
| A309 G-2F | TGAGGACGGTTATGTCTGGAGGTGCTCCGTTAGGGAAA |
| G334A-1R | CCCTGCCTCTGTCATTGCATATCCCTGACCAAGTTTGG |
| G334A-2F | CCAAACTTGGTCAGGGATATGCAATGACAGAGGCAGGG |
| K441A-1R | TTGGAAGCCTTTGTATGCGATTATTTCCTTTAGTCTAT |
| K441A-2F | ATAGACTAAAGGAAATAATCGCATACAAAGGCTTCCAA |
| Q446A-1R | TCAGCAGGGGCTACTGCGAAGCCTTTGTATTTGATTA |
| Q446 A-2F | TAATCAAATACAAAGGCTTCGCAGTAGCCCCTGCTGA |
| K526A-1R | GTCCTTTCGAAGAATCGCTCCAGATGGTGATTTTGGA |
| K526A-2F | TCCAAAATCACCATCTGGAGCGATTCTTCGAAAGGAC |
| Q-109957-F | TCCACCTGTTGTGCCACACT |
| Q-109957-R | TTAATCCTGATGACGCGGTG |
| Q-20539-F | TTGCTGTAAGCCTCGGGAGA |
| Q-20539-R | ACCCTCATCACCCTGCACTC |
| Q-20936_c0-F | TGCTCAATTGCAAAGGGGAA |
| Q-20936_c0-F | GCCAACGCAAGTACAATCGG |
| Q-20936_c1-F | TAACCAAGGTCGCCAGTATGC |
| Q-20936_c1-R | TCCTCCTAAGCAGTTGGGTGA |
| Q-21343-F | GTGGTGGAAGGGTTTGTGGA |
| Q-21343-R | CTCCTGCTTTCCTCCAACGA |
| Q-269041-F | ACCAGGTGCATGTGTGGTGA |
| Q-269041-R | TCCCCGAAGATGATTTTGGA |
| Q-27540-F | TCCCCTGGGAAAGGAAATCA |
| Q-27540-R | ACCGAACCCCAGTGCAAACT |
| Q-28765-F | TATCAGAGCAGGTTGCGCCT |
| Q-28765-R | AGCTCGGACTTGCTCAACGA |
| Q-28824-F | AAAGGTTTCCAAGTTGCCCC |
| Q-28824-R | GGTGGAACCATTTGATCGCA |
| Q-31594_c0-F | TTATGGAGCGGCTTGAGGAA |
| Q-31594_c0-R | CTTGACCCACAGGTTTCCCA |
| Q-31594_c2-F | TAATTCGAGTCCGAGCCACG |
| Q-31594_c2-R | GCTTGAGCCGGTTCATTTCC |
| Q-31594-F | CTTCTTGGGCACTTTGAAAGC |
| Q-31594-R | GGCGAAGAGATAAACTGTGCG |
| Q-36048-F | CATCATCAGGCGATTTTCGC |
| Q-36048-R | TCTCAGTCCGATACGGCTGC |
| Q-398667-F | GTCTTGGTTCTTGTCTGTTTTGG |
| Q-398667-R | TTCGTCTTTTTGTTTGATCAACC |

**Table S2. Accession numbers and the names of the species used in this study**

| Accession number | Species | Gene |
| --- | --- | --- |
| AMP18194.1 | *Angelica sinensis* | 4CL1 |
| P14912.1 | *Petroselinum crispum* | 4CL1 |
| AIT52347.1 | *Daucus carota* | 4CL2 |
| NP_001312667.1 | *Nicotiana tabacum* | 4CL1 |
| AAP68991.1 | *Salvia miltiorrhiza* | 4CL2 |
| AEO52694.1 | *Petunia x hybrida* | 4CL1 |
| NP_001312554.1 | *Nicotiana tabacum* | 4CL2 |
| AAC39366.1 | *Populus trichocarpa* | 4CL1 |
| BAD90937.1 | *Scutellaria baicalensis* | 4CL1 |
| AFC89540.1 | *Populus tomentosa* | 4CL4 |
| AFC89541.1 | *Populus tomentosa* | 4CL5 |
| AAL56850.1 | *Populus tomentosa* | 4CL1 |
| AAA82888.1 | *Arabidopsis thaliana* | 4CL1 |
| AEE76480.1 | *Arabidopsis thaliana* | 4CL2 |
| AEE34323.1 | *Arabidopsis thaliana* | 4CL3 |
| NP_001236418.1 | *Glycine max* | 4CL1 |
| NP_001236236.1 | *Glycine max* | 4CL2 |
| Q6ZAC1.1 | *Oryza japonica* | 4CL5 |
| Q67W82.1 | *Oryza japonica* | 4CL4 |
| Q6ETN3.1 | *Oryza japonica* | 4CL3 |
| Q42982.2 | *Oryza japonica* | 4CL2 |
| P17814.2 | *Oryza japonica* | 4CL1 |
| NP_201143.1 | *Arabidopsis thaliana* | 4CL-like9 |
| AFD33352.1 | *Cannabis sativa* | AAE8 |
| NP_190468.1 | *Arabidopsis thaliana* | 4CL-Like10 |
| XP_011076162.1 | *Sesamum indicum* | OCL |
| AHM88425.1 | *Fraxinus mandshurica* | 4CL13 |
| XP_002510783.1 | *Ricinus communis* | OCL |
| NP_001311686.1 | *Capsicum annuum* | OCL |
| XP_009803076.1 | *Nicotiana sylvestris* | 4CL-like10 |
| XP_009601805.1 | *Nicotiana tomentosiformis* | 4CL-like10 |
| XP_016547391.1 | *Capsicum annuum* | 4CL-like7 |
| XP_016476896.1 | *Nicotiana tabacum* | 4Cl-like 7 |
| AHL44983.1 | *Fraxinus mandshurica* | 4CL4 |
| XP_009786809.1 | *Nicotiana sylvestris* | 4Cl-like 7 |
| ADG46006.1 | *Isatis tinctoria* | 4CL |
| AFD33347.1 | *Cannabis sativa* | AAE3 |
| KX254614 | *Peucedanum praeruptorum* | 4CL1 |
| KX254613 | *Peucedanum praeruptorum* | 4CL7 |
| KX254612 | *Peucedanum praeruptorum* | 4CL10 |
| Q9LU36.1 | *Arabidopsis thaliana* | 4CL5 |
| Q9M0X9.1 | *Arabidopsis thaliana* | 4CL6 |
| Q84P24.2 | *Arabidopsis thaliana* | 4CL7 |
| Q84P21.2 | *Arabidopsis thaliana* | 4CL9 |
| Q9LQ12.1 | *Arabidopsis thaliana* | 4CL10 |
| Q84P26.2 | *Arabidopsis thaliana* | 4CL11 |

**Table S3.** **Dominant ions in high resolution mass spectra of hydroxycinnamate CoA thioesters in positive mode**

| Compound | Mr | ESI positive m/z of [M+ H]+/ [M +2H]^2+^ |
| --- | --- | --- |
| *p*-Coumaroyl-CoA | 913.7 | 914.1597/ 457.5840 |
| Feruloyl-CoA | 943.7 | 944.1707/ 472.5909 |
| Caffeoyl-CoA | 929.7 | 930.1548/ 465.5814 |
| Cinnamoyl-CoA | 897.7 | 898.1635/ 499.5882 |
| Isoferuloyl-CoA | 943.7 | 944.1704/ 472.5895 |
| *o*-Coumaroyl-CoA | 913.7 | 914.1580/ 457.5845 |

**Table S4.** **Relative activities (%) of Pp4CL1 mutants towards *p*-coumaric acid, ferulic acid, caffeic acid and isoferulic acid.** The activity of the enzyme towards *p*-coumaric acid, ferulic acid, caffeic acid and isoferulic acid are set as 100%, respectively.

|  | | *p*-Coumaric acid | Ferulic  acid | Caffeic  acid | Isoferulic  acid |
| --- | --- | --- | --- | --- | --- |
| WT | Pp4CL1 | 100.0% | 100.0% | 100.0% | 100.0% |
| M1 | Y239A | 88.5% | 0.0% | 43.2% | 64.9% |
| M2 | Y239F | 86.4% | 83.7% | 36.3% | 92.8% |
| M3 | Y239W | 0.0% | 0.0% | 32.2% | 3.9% |
| M4 | A243S | 56.9% | 0.0% | 15.2% | 82.0% |
| M5 | M306K | 46.1% | 72.2% | 27.5% | 34.7% |
| M6 | M306A | 0.0% | 0.0% | 31.9% | 15.6% |
| M7 | G308A | 38.4% | 0.0% | 30.0% | 24.4% |
| M8 | A309G | 103.1% | 62.9% | 19.6% | 87.4% |
| M9 | G334A | 0.0% | 0.0% | 20.0% | 14.8% |
| M10 | K441A | 52.0% | 35.0% | 28.6% | 72.7% |
| M11 | Q446A | 103.1% | 39.1% | 110.7% | 88.3% |
| M12 | K526A | 73.0% | 33.3% | 28.4% | 57.7% |


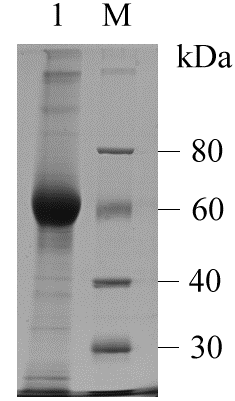


**Figure S1. SDS-PAGE analysis of recombinant His_6_-Pp4CL1 purified by affinity chromatography**. Lane M: Protein Marker; Lane 1: His-tagged Pp4CL1 (predicted M.W., 59.7 kDa) purified on Ni Sepharose.


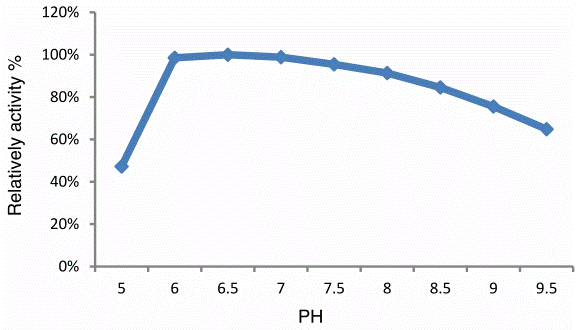


**Figure S2. Effects of various pHs on the activity of Pp4CL1**


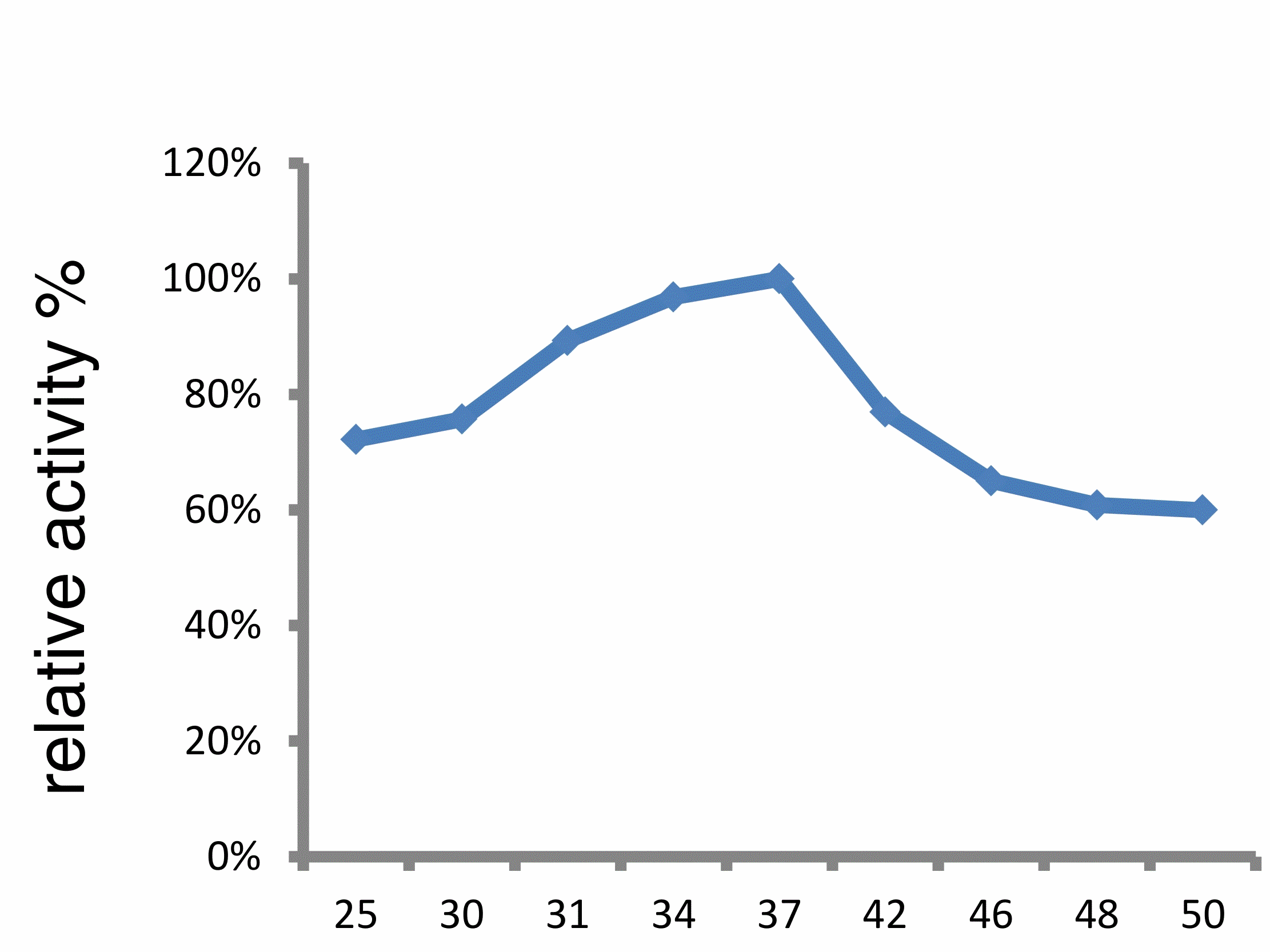


**Figure S3. Effects of various temperatures on the activity of Pp4CL1**


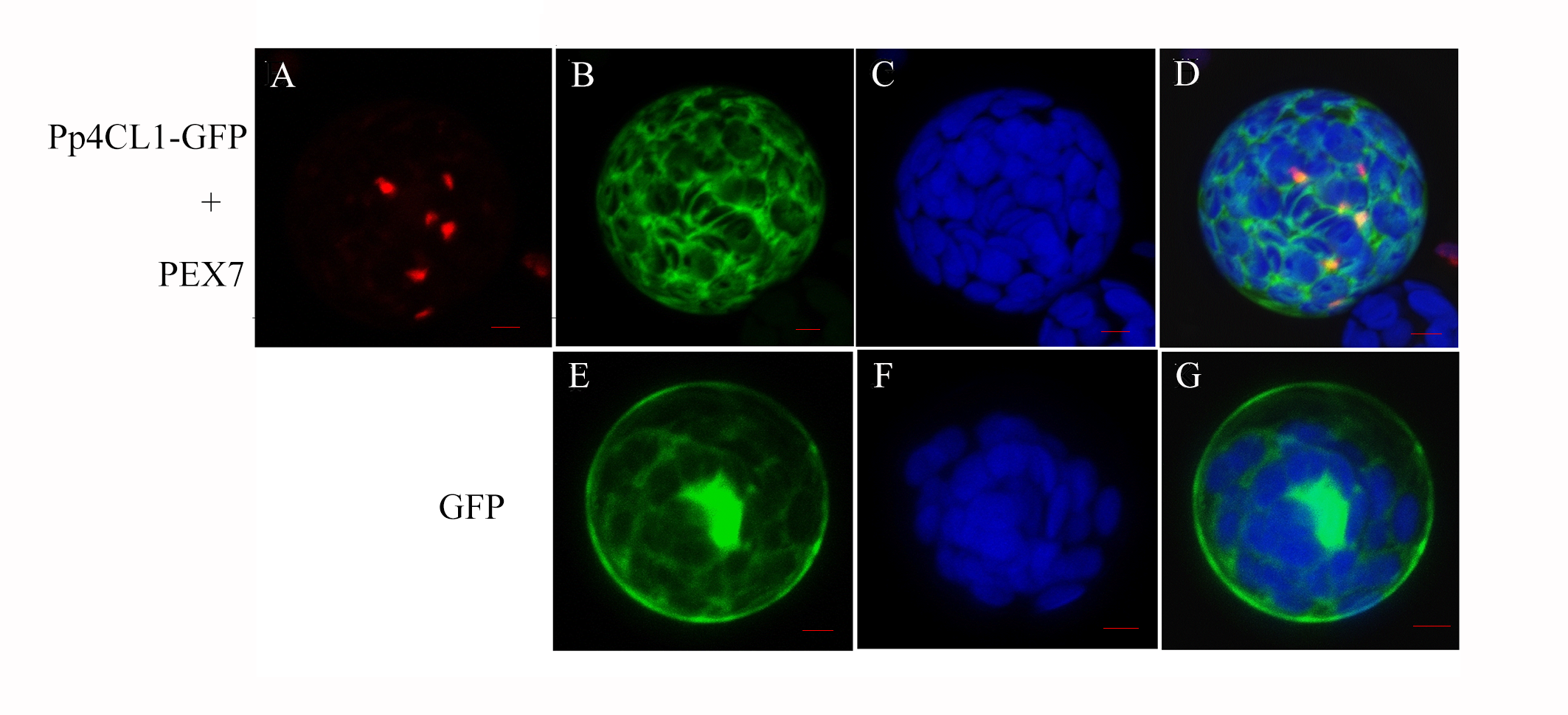


**Figure S4. Subcellular localization of Pp4CL1 protein with additional peroxisomal marker (PEX7). GFP fluorescence is shown in green and chlorophyll autofluorescence is shown in blue. In (A), fluorescence of PEX7 (peroxin 7) used as a peroxisomal marker is shown in red. Merged panel shows combined fluorescence from GFP, chloroplasts, and peroxisomes. Bars = 20μm.**
